# The influence of passive heating garments worn in temperate and cold conditions prior to simulated performance for male soccer substitutes

**DOI:** 10.14814/phy2.70189

**Published:** 2025-02-21

**Authors:** Gavin Cowper, Stuart Goodall, Kirsty M. Hicks, Louise Burnie, Kai Fox, David Duffy, Marc A. Briggs

**Affiliations:** ^1^ Faculty of Health and Life Sciences Northumbria University Newcastle Upon Tyne UK; ^2^ Physical Activity, Sport and Recreation Research Focus Area, Faculty of Health Sciences North‐West University Potchefstroom South Africa; ^3^ Performance, Medical and Innovation Department, Washington Spirit Soccer Club Washington DC USA

**Keywords:** passive heating, performance, soccer, thermoregulation

## Abstract

Lengthy periods of inactivity are experienced by substitutes during a soccer match, which can decrease muscle temperature, ultimately impacting performance. This study aimed to determine the effects of using a passive heat intervention in both a cold (2°C) and thermoneutral (18°C) environment on simulated soccer performance and perceptual responses. On four occasions, 14 trained male players, completed a pre‐match warm‐up, followed by 45 min of rest. After, players completed a half‐time re‐warm‐up, followed by an additional 15 min of rest, simulating 60 min as a substitute. During these periods, players wore tracksuit bottoms (CON), or heated trousers (HEAT), over soccer attire. Once 60 min concluded, participants performed a Soccer Match Simulation (SMS) to assess physical performance. HEAT improved 15 m sprint performance in 2°C (2.8%; *p* < 0.001) and 18°C (2.6%; *p* < 0.001) conditions. Further, in HEAT, a significant trial and time effect on countermovement jump height and repeated sprint performance was observed in both 2 and 18°C. Upon match entry, participants felt warmer (*p* < 0.01), more comfortable (*p* < 0.01), and felt an increase in match readiness following HEAT, during both conditions. Applying heated garments before match entry for soccer substitutes positively impacts physical performance and match readiness in thermoneutral and cold environments.

## 1 INTRODUCTION

Prior to a soccer match, a warm‐up routine has been suggested to be an imperative factor in task readiness with the anticipation that it will enhance performance. A key benefit of an active warm‐up is the increase in muscle temperature, which results in various physiological benefits, including an increased speed of contraction and relaxation of muscle fibers, increased anaerobic metabolic capacity and nerve conduction enhancements in both the peripheral and central nervous system (Mohr et al., 2004). However, lengthy transition periods occur between an active warm‐up and exercise performance, which decline muscle temperature, thereby reducing performance capacity (Cowper et al., 2022; Faulkner, Ferguson, Gerrett, et al., 2013; Faulkner, Ferguson, Hodder, & Havenith, 2013). As such, methods are needed to assist athletes during transition periods, to maintain the benefits of a warm‐up with the aim of optimizing performance.

The application of passive heating garments has been shown to reduce the decline in muscle and core temperature during lengthy transition periods and has subsequently been found to enhance specific sporting performances (Cowper et al., 2021; Faulkner, Ferguson, Gerrett, et al., 2013; Faulkner, Ferguson, Hodder, & Havenith, 2013). However, limited studies have examined the effects of long‐duration performance (>5 min) using passive heating devices, and this is likely because previous studies using hot showers/baths have found a detrimental effect on long‐duration performance at ambient temperatures (Gregson et al., 2002a, 2002b). During long‐duration activity in thermoneutral environments, the detrimental effects caused by passive heat during the transition period have been reported to be due to lower heat‐storage capacity and earlier attainment in critical core temperature (Fortney et al., 1984; Nadel, 1987). However, in below‐ambient temperatures, where lower body temperatures are experienced, might increase the time that the body takes to reach a critical core temperature during long‐duration performance. This outcome could lead to passive heating being beneficial in preventing bodily temperature from dropping too low and therefore maintaining endurance performance (Cowper et al., 2021; Marino, 2002).

Fairbank et al. (2021) found small improvements in high‐intensity running performance during a 22‐min simulated playing bout for interchanged rugby league players when a knee‐length sub‐suit jacket was utilized for 15 min in “changing room” conditions (~19.9°C) between active warm‐up and simulated playing bout, with warm‐up and performance taking place in ~12°C. The sole study which has examined the effects on performance when utilizing passive heat, during a transition period at below ambient temperatures (8°C), reported a significant improvement in rowing performance (Cowper et al., 2021). Therefore, further research on whether passive heating strategies could be applied to sports whereby it is difficult to maintain muscle temperature from metabolic heat production alone is imperative. More specifically, sports with limited opportunity to conduct sport‐specific active rewarming with lengthy transition times in below ambient temperatures, such as substitutes in game‐based activities. Substitutes in soccer squads typically have a lengthy duration of up to 45 min of passive rest, interchanged with brief periods of submaximal intensity warm‐ups between the pre‐match or half‐time warm‐up and match entry. Additionally, the majority of English Premier League replacements were made at half‐time or between min 60–85 (Bradley et al., 2014). During this period, there is an increased risk of reduced core and muscle temperature (Hills et al., 2021), which would be exaggerated in below‐ambient temperatures, especially relevant with common mid‐afternoon and evening kick‐off times in the United Kingdom during winter months. Thus, methods are needed to support soccer substitutes in maintaining muscle activation and raised muscle temperature during such transition periods, so once called into match‐play, players can still capitalize on the benefits of increased bodily temperature. Therefore, practical methods of passive heating could be used during transition periods to counter the decline in muscle temperature and subsequently improve performance.

Cowper et al. (2023) found that following the application of heating garments, throughout unused soccer substitute simulation, muscle temperature maintenance was improved throughout long transition periods (45 min) in thermoneutral (18°C) and cold environments (2°C). Therefore, the combination of a heat maintenance device during the transition period between warm‐up and competition may yield performance‐enhancing benefits for soccer performance. Although physical performance does not guarantee success (Di Salvo et al., 2009; Lago‐Peñas et al., 2010), soccer substitute entry has been deemed to be effective from a physical capacity (Bradley et al., 2014; Hills et al., 2018).

Accordingly, this study aimed to determine whether the use of heated garments during the transition period before match entry for soccer substitutes can enhance thermophysiological functions, subjective readiness and aspects of soccer performance in both thermoneutral (18°C) and cold (2°C) environments.

## 2 METHODS AND MATERIALS

### 2.1 Participants

Fourteen trained male soccer players (mean ± SD, age: 29 ± 6.2 years, stature: 181.7 ± 5.2 cm, body mass: 78.1 ± 9.2 kg, estimated maximal oxygen uptake [V̇O_2max_]: 50.1 ± 2.4 mL kg^−1^ min^−1^) participated in this study. The population was defined as soccer players who are currently playing regular soccer at semi‐professional or high‐level university standard. 14 participants were deemed sufficient when calculating sample size, using a change in mean 30 m sprint performance, a crossover design in a similar population, and the SD of non‐tapered performance times (± 0.04 s), a statistical power of 0.8 and a mean difference in 30 m sprint performance 0.07 s, which had an ES of 1.75 based on findings (Mohr et al., 2004) (G*power 3.1, University of Duesseldorf, Germany). None of the participants had supplemented their diets with any putative ergogenic aid and were free of injury for 6 months before the start of the study. All participants were explained the experimental procedures, potential benefits, and associated risks, before providing informed consent to participate. Participants avoided the consumption of caffeine and alcohol and refrained from any vigorous exercise 24 hours before all testing. Participants were also asked to emulate their food consumption during the study. Prior to any experimental activity, the study was approved by Northumbria University ethics committee (#45401) and all procedures were conducted according to all aspects of the Declaration of Helsinki, apart from registration in a database.

### 2.2 Experimental design

This study used a within‐participant, randomized, repeated measured experimental design. Each participant was required to visit the environmental chamber (TIS Services, Alton, Hampshire, UK) on five separate occasions including one familiarization visit, with each session ~7 days apart. Trials were performed at the same time of day (±1 hour) to minimize circadian effects. For all participants, the order of the experimental visits was randomized using simple randomization via the form of random number generation software (GraphPad Prism 9, GraphPad Software, USA). During the four experimental visits, upon arrival, participants completed a 15‐min standardized soccer warm‐up on an indoor running track (~18°C) (Fashioni et al., 2020), then entered an environmental chamber for 45 min of passive rest, simulating the first half for a soccer substitute, in either of two environmental conditions: (1) replicating an average UK ambient temperature (18°C), (2) the common temperature experienced during evening kick‐off times during winter months in the United Kingdom (2°C). In each environmental condition, during the passive substitute simulation, participants wore a standardized tracksuit top and either a pair of standardized tracksuit bottoms (CON) or, a pair of externally heated trousers (HUUB Design, Derby, UK) over standard soccer clothing during the HEAT intervention. Therefore, the four conditions were HEAT in 18°C (HEAT18), CON in 18°C (CON18), HEAT in 2°C (HEAT02), and CON in 2°C (CON02). Following this, tracksuits were removed, and participants returned to the indoor running track to complete a 15‐min half‐time re‐warm‐up, then returned to the chamber for an additional 15 min to simulate minute 45–60 min of a soccer match. After the 60 min, participants returned to the indoor running track to complete a 30‐min modified Soccer Match Simulation (SMS) and a pre, 15‐min and post SMS testing block (Briggs et al., 2017).

### 2.3 Procedures

#### 2.3.1 Familiarization session

Prior to the four experimental sessions, the protocol was explained, and initial measurements were taken (age, stature, and body mass). Subsequently, participants performed a Yo‐Yo Intermittent Recovery Test 2 to estimate V̇O_2max_ (Bangsbo et al., 2008), to ensure speeds of the SMS were relative to aerobic capacity (Briggs et al., 2017). Furthermore, participants had the opportunity to become familiar with the testing blocks and the SMS that were performed during experimental sessions.

#### 2.3.2 Substitute simulation

Upon arrival, participants were dressed in typical soccer match day apparel and a heart rate (HR) monitor was fitted around the chest (Polar FT1; Polar Electro155 Oy, Kempele, Finland). Once the participants were prepared for exercise, they completed a 15‐min soccer‐specific warm‐up on the 50 m indoor running track to best replicate typical pre‐match practice. The 15‐min protocol included a combination of bodyweight exercises, as well as ballistic, and plyometric movements. It was performed over a 20 m circuit utilizing a raise, activate, mobilize, and potentiate method (Jeffreys, 2007). The participants were instructed to perform two sets of jogging (40 m) and skipping (40 m), followed by one set (20 m) of each dynamic stretch exercise (knee raises; heel flicks; lateral side lunges; front lunges; high kicks; tuck jumps; and reactive sprints). The movements were performed in tandem with an example being provided by the lead researcher. Following the completion of the dynamic stretches, participants completed two sets each of straight sprinting, tuck jump into sprints, and reactive sprints (facing the opposite way and then turning and sprinting) (Fashioni et al., 2020).

Following the active warm‐up, participants then completed a baseline testing block. Participants then entered the environmental chamber and remained seated. A handheld aural thermistor (Grant Instruments, Cambridge, UK) was inserted into the ear canal to monitor tympanic temperature (T_tymp_). The aural thermistor was placed outside of the chamber at room temperature (~18°C) when it was not in use and passed in through a service hatch for measurements to be taken. Participants were then seated for 45 min, simulating substitute activity for the 1st half of a soccer match. After the 45 min of passive rest, participants returned to the track and performed a 15‐min re‐warm‐up, simulating the half‐time period. Participants performed light ball work for the initial 5 min; in the following 7 min, participants performed the soccer‐specific aerobic field test agility course, involving repeated 20 m soccer‐specific runs, targeting to maintain 70% HR_Max_ (Edholm et al., 2014; Lovell et al., 2013; Mohr et al., 2004), allowing 3 min to travel to and from the chamber. Following the re‐warm‐up, participants entered the environmental chamber and were again instructed to remain seated for an additional 15 min, replicating being a soccer substitute from minute 45 to 60 in the second half of a soccer match.

Throughout these passive rest phases, participants wore either HEAT or CON. The heated trousers (HUUB Design, Derby, UK) were chosen because of the optimal coverage of the quadriceps, hamstrings, calves, and glutes with the heating elements in comparison to other varieties (Faulkner, Ferguson, Gerrett, et al., 2013). The heating elements stretch panels allow for optimal heat transfer, as the material is maintained close to the body, thus decreasing convection, while allowing movement. The maximum temperature of the heating elements was 45°C but skin temperature is known to be lower (Wilkins & Havenith, 2017). Based on similar heated trousers, when the heating elements were inactive (CON), the insulation value was approximately 0.559 m2·K·W^−1^ (3.6 clo) for the legs and hips in isolation that were covered by the garment. When the heating elements were initiated (HEAT), insulation of the legs and hips increased to give a local insulation value of approximately 0.842 m2·K·W–1 (5.4 clo) (Faulkner, Ferguson, Hodder, & Havenith, 2013). During the passive rest periods, T_tymp_ as well as thermal comfort (TC), and sensation (TS) were monitored every 15 min (Gagge et al., 1967). Furthermore, following the pre‐match warm‐up, half‐time re‐warm‐up, and immediately prior to match entry, participants completed the “Elite Performance Readiness Questionnaire” EPRQ; (Dean et al., 1990), which took 30–60 s to complete. After the 15 min of passive rest, players performed a 2‐min low‐intensity warm‐up while retuning to the track and tracksuits were removed to perform the SMS protocol (Russell, Rees, et al., 2011).

### 2.4 Performance testing

#### 2.4.1 Testing blocks

Participants' countermovement jump (CMJ) height and 30‐m repeated sprint maintenance (RSM) were tested at four‐time points (baseline, pre‐SMS, post SMS block 2 and post‐SMS), each required participants to perform three CMJ's separated by 10 s of passive recovery and three 30 m sprints separated by 25 s of active recovery (light jogging). In both performance tests, the mean value of the three attempts were used for analysis. CMJ height was determined using an optical measuring system (OptoJump Next, Microgate Corp, Italy). Players began each repetition from a standing position and performed a preparatory crouching action (at a consistent, self‐determined level) before explosively jumping out of the dip for maximal height. It was recommended that at take‐off the participants leave the floor with the knees and ankles extended and land in a similarly extended position (Glatthorn et al., 2011). Hands were isolated at the hips for the entire movement to eliminate any influence of arm swing. For RSM testing, players commenced each repetition from a standing start at a distance of 0.3 m behind the start line. Sprint times were recorded by using two pairs of timing gates with infrared light sensors, having a precision of 0.01 s (Brower Timing, Utah); verbal encouragement was provided throughout each attempt. Additionally, to avoid pacing, participants performed the trials alone. The test–retest reliability of the CMJ (Coefficient of variation [CV]: 2.7%) and RSM (CV: 1.7%) for this study has been established.

#### 2.4.2 Soccer match simulation

**FIGURE 1:**
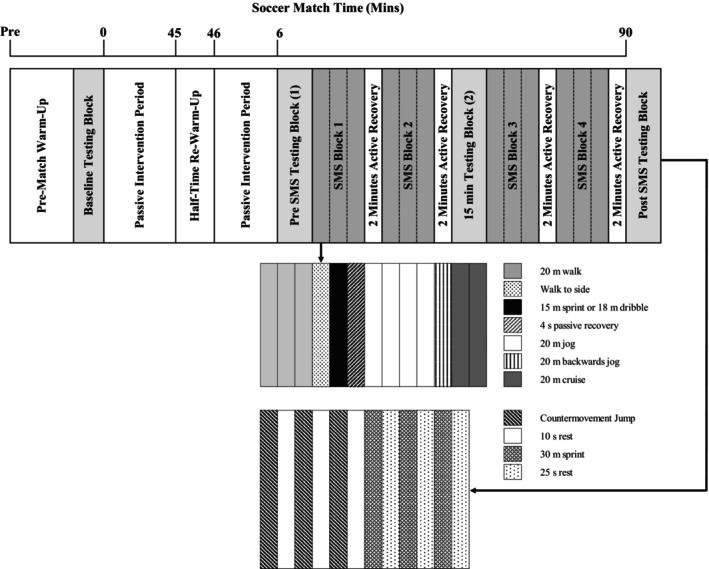
Soccer Match Simulation timeline.

A 30‐min, modified version of the SMS was implemented to monitor the physical and skill performance of soccer substitutes to simulate entering a soccer match at 60 min. The SMS was ~30 min in length and was made up of ~4.5‐min blocks consisting of three repeated cycles of three 20 m walks, one walk to the side (∼1 m), an alternating 15 m sprint or an 18 m dribble test, a 4‐s passive recovery period, five 20 m jogs at a speed corresponding to 40% V̇O_2max_, one 20 m backward jog at 40% V̇O_2max_, and two 20 m strides at 85% V̇O_2max_. A 2‐min recovery period followed all blocks of exercise (see Figure 1).

Four blocks of intermittent exercise and skill testing were completed during each main trial and participants covered a total distance of approximately 3.3 km while performing 6 maximal 15 m sprints and 6 dribbles. Fifteen meter sprints and 18 m dribbles (assessed for precision and average speed) were recorded throughout the SMS. Players were required to dribble the ball as fast and as accurately as possible between cones spaced every 3 m (Russell, Benton, & Kingsley, 2011). All dribbles were video recorded (50 Hz; 103 DCRHC96E; Sony Ltd., UK) and digitisation processes (Kinovea version 0.8.15; Kinovea Org., France) derived speed (time taken to complete the distance) and precision (distance of the ball from each cone) data were recorded. The test–retest reliability for all components of the SMS have been established, including performance (CV: 2.1%), physiological (CV: 2.6%) and metabolic (CV: 16.1%) responses (Russell, Rees, et al., 2011). The repeatability of the original 90‐min SMS and responses to this exercise protocol has previously been determined (Harper et al., 2016; Russell, Rees, et al., 2011).

#### 2.4.3 Perceptual measures

TC and TS were measured using visual analogue scales and were taken throughout the passive protocols (Gagge et al., 1967). The number range for both scales was consistent, but anchors varied (TC, −3 very uncomfortable, −2 uncomfortable, −1 just uncomfortable, 0 neutral, 1 just comfortable, 2 comfortable, 3 very comfortable: TS, −3 cold, −2 slightly cold, −1 cool, 0 neutral, 1 warm, 2 slightly hot, 3 hot). Participants were asked to ensure the trousers felt “comfortable (≤2)” and “hot (≤3),” if the participant felt “uncomfortable (≥ − 2)” the heat stimulus was reduced. If participants felt “cold (≥3),” extra clothes including a thick hooded Winter jacket and gloves were provided which were replicated for each environmental condition (nine participants required extra clothes at 13.3 ± 6.1 min in the 2°C environment), which was replicated for each half of the trial and for each individual environmental condition.

#### 2.4.4 Data analysis

All statistical tests were processed in IBM SPSS Statistics 27 (SPSS Inc., Chicago, IL). Parameters measured throughout the passive rest period including TS, TC, and T_tymp_ were analyzed (two‐tailed) following the first half using a two‐way (trial [2] × time [4]) analysis of variance (ANOVA) and second half using a two‐way (trial [2] × time [2]) ANOVA with multiple comparisons followed up by using the Tukey method when significant main or interaction effects were observed.

Furthermore, testing block performance measures including CMJ height and 30 m RSM were analyzed using a two‐way (trial [2] × time [4]) and SMS performance indicators, including dribbling (precision and speed) and sprint velocities (15 m) were analyzing using a two‐way (trial [2] × time [6]) ANOVA with multiple comparisons followed up by using the Tukey method when significant main or interaction effects were observed. The accepted level of significance was *p* < 0.05. Data were presented as mean ± SD.

## 3 RESULTS

### 3.1 2°C condition

#### 3.1.1 Performance

##### Testing blocks

**FIGURE 2:**
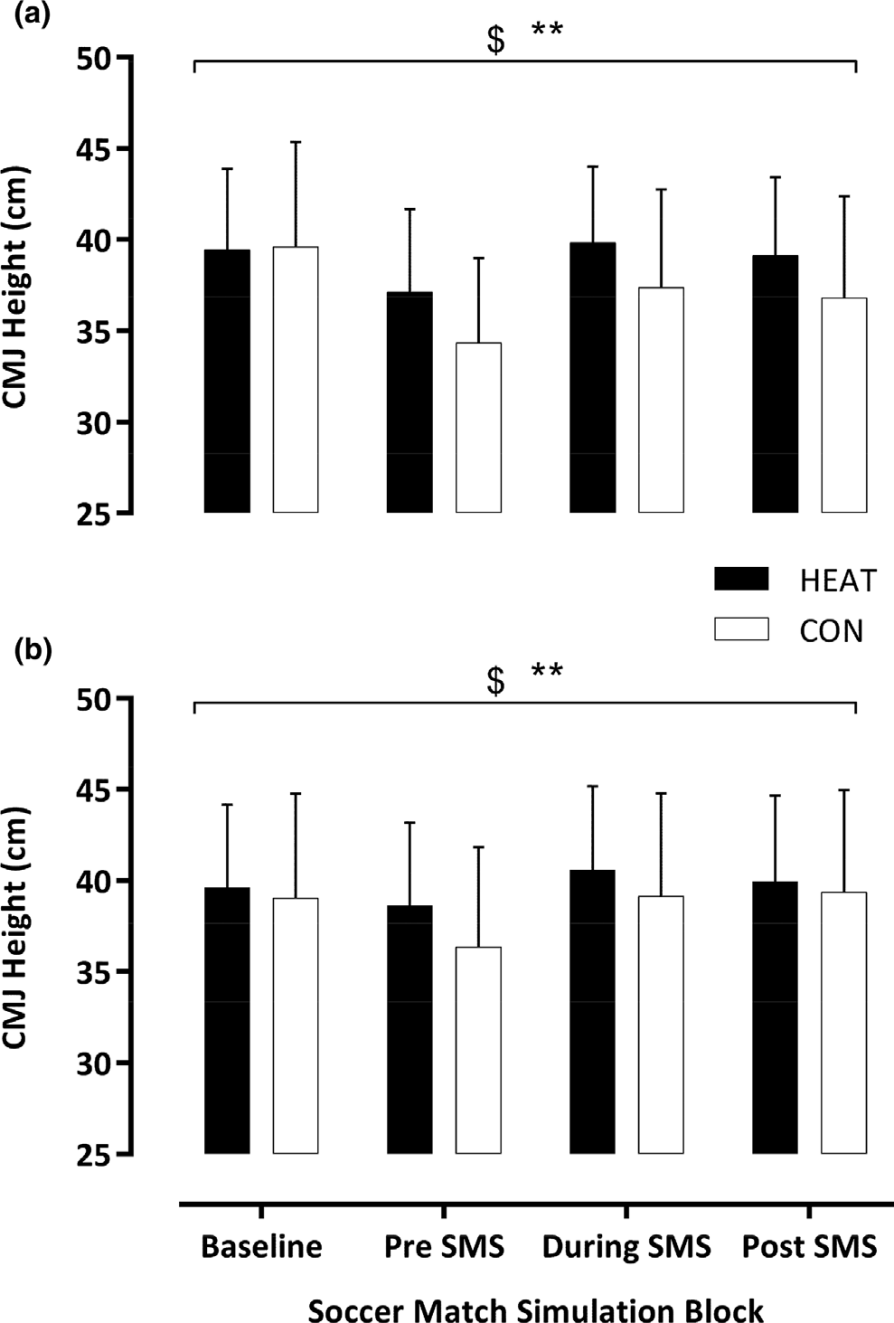
Measurement of CMJ Height (cm) after the passive intervention in 2°C (a) and 18°C (b) following a pre‐match warm‐up (Baseline), pre‐SMS after the 60‐min substitute simulation, during the SMS at minute 15 and post‐SMS. Data presented as mean ± SD. $ denotes a time effect, ** denotes condition effect (*p* < 0.05).

**FIGURE 3:**
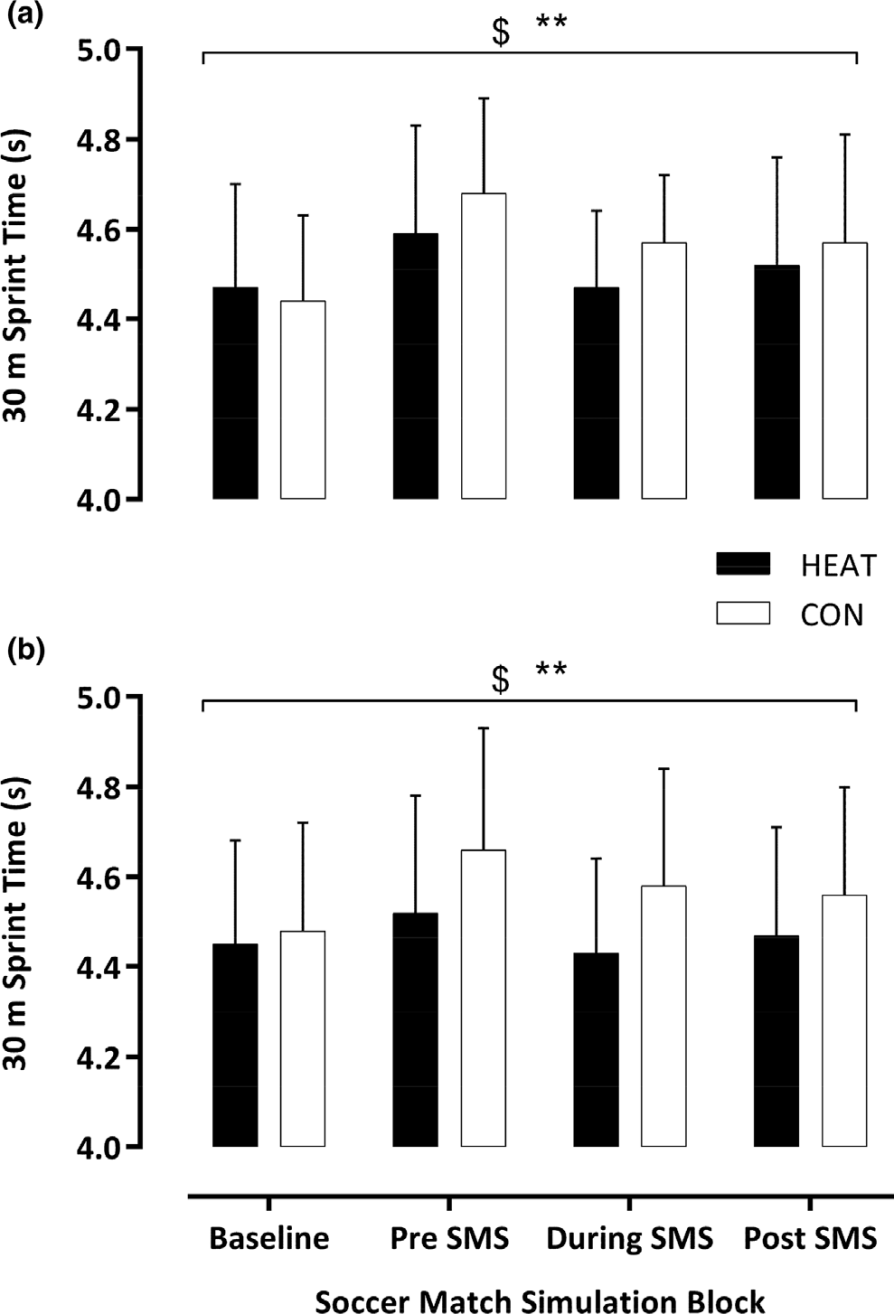
Measurement of average 30 m sprint time (s) after the passive intervention in 2°C (a) and 18°C (b) following a pre‐match warm‐up (Baseline), pre‐SMS after the 60 min substitute simulation, during the SMS at minute 15, and post‐SMS. Data presented as mean ± SD. $ denotes a time effect, ** denotes condition effect (*p* < 0.05).

The pre‐match warm‐up elicited similar CMJ (HEAT: 39.4 ± 4.4 vs. CON: 39.6 ± 5.8 cm; *F*_1,13_ = 0.052; *p* = 0.82) and 30 m RSM (HEAT: 4.46 ± 0.24 vs. CON: 4.44 ± 0.19 s; *F*_1,13_ = 0.65; *p* = 0.44) values compared to baseline. Throughout the SMS, CMJ (*F*_3,11_ = 27, *p* < 0.001) and RSM (*F*_3,11_ = 13.94, *p* < 0.001) reduced over time but were enhanced in HEAT versus CON for CMJ (*F*_1,13_ = 9.27, *p* = 0.009) and RSM (*F*_1,13_ = 5.13, *p* = 0.041). Further, the rate of change was attenuated in CMJ (*F*_3,11_ = 8.1, *p* = 0.004) and RSM (*F*_3,11_ = 10.5, *p* = 0.001). Following a 60‐min substitute simulation, post hoc analyses showed no difference in CMJ or RSM (Figure 2a & 3a).

##### Soccer match simulation

Following the application of HEAT versus CON, throughout the SMS, mean 15 m sprint improved (2.53 ± 0.11 vs. 2.60 ± 0.14 s; *F*_1,13_ = 23.01; *p* < 0.001). However, 15 m sprint time was not reduced over time. Throughout the SMS, dribbling speed (5.76 ± 0.74 vs. 5.89 ± 0.72 s; *F*_1,13_ = 2.595; *p* = 0.13) and precision (31.41 ± 3.43 vs. 31.11 ± 3.56; *F*_1,13_ = 0.11; *p* = 0.74) were comparable between HEAT and CON. Further, the rate of change in dribbling speed and precision did not differ over time (*p* ≥ 0.072).

### 3.2 Perceptual response

#### 3.2.1 Thermal Comfort and Thermal Sensation

Perceptual measures were similar at baseline in each condition. Throughout the simulated first half, TC (*F*_3,39_ = 39.357, *p* = 0.002) and TS (*F*_3,39_ = 72.596, *p* < 0.001) reduced over time and the decline was attenuated in HEAT versus CON in TC (*F*_1,13_ = 29.51, *p* < 0.001) and TS (*F*_1,13_ = 43.62, *p* < 0.001). Further, the rate of change in TC (*F*_3,39_ = 20.02, *p* < 0.001) and TS (*F*_3,39_ = 9.01, *p* < 0.001) differed over time between conditions and post hoc analyses confirmed that participants felt “more comfortable” and “warmer” after 15 (TC: *p* = 0.003; TS: *p* < 0.001), 30 (TC: *p* < 0.001; TS: *p* < 0.001), and 45 min (TC: *p* < 0.001; TS: *p* < 0.001) in HEAT versus CON. During the simulated second half, TC (*F*_1,13_ = 25.138, *p* < 0.001) and TS (*F*_1,13_ = 19.158, *p* < 0.001) reduced over time and the decline was attenuated in HEAT versus CON in TC (*F*_1,13_ = 25.83, *p* < 0.001) and TS (*F*_1,13_ = 44.807, *p* < 0.001). Further, the rate of change in TC (*F*_1,13_ = 10.864, *p* = 0.006) and TS (*F*_1,13_ = 12.621, *p* = 0.004) differed over time between conditions and post hoc analyses confirmed that participants felt “more comfortable” and “warmer” after minute 60 (TC: *p* < 0.001; TS: *p* = 0.001) (Table 1).

**TABLE 1:** Thermal comfort and thermal sensation throughout the passive rest intervention periods.

Condition	PWU	15	30	45	PWU	60
Thermal comfort (TC)						
2°C CON	1.0 ± 1.2	0.0 ± 1.4	−0.8 ± 1.5	−1.6 ± 1.4	1.3 ± 0.6	−0.9 ± 1.2
2°C HEAT	1.1 ± 1.1	1.4 ± 0.9**	1.2 ± 0.9**	0.5 ± 1.0**	1.4 ± 0.6	0.9 ± 1.0**
18°C CON	1.2 ± 1.0	1.4 ± 0.9	1.4 ± 0.9	1.5 ± 0.9	1.2 ± 0.8	1.6 ± 0.5
18°C HEAT	1.1 ± 1.3	1.9 ± 1.0	2.4 ± 0.6**	2.4 ± 0.6**	1.2 ± 1.3	2.3 ± 0.5**
Thermal sensation (TS)						
2°C CON	1.1 ± 0.9	−0.9 ± 0.9	−1.5 ± 1.1	−2.4 ± 0.6	1.2 ± 1.0	−1.6 ± 1.2
2°C HEAT	1.5 ± 0.7	0.9 ± 0.7**	0.4 ± 1.0**	−0.2 ± 1.2**	1.5 ± 0.8	0.1 ± 1.3**
18°C CON	1.6 ± 0.8	0.4 ± 0.7	0.2 ± 1.0	0.1 ± 0.8	1.6 ± 0.5	0.5 ± 0.8
18°C HEAT	1.7 ± 0.7	1.1 ± 0.7**	1.2 ± 0.8**	1.2 ± 0.9**	2.1 ± 0.7	1.5 ± 0.7**

*Note*: Visual analogue scale anchors: TC, −3 very uncomfortable, −2 uncomfortable, −1 just uncomfortable, 0 neutral, 1 just comfortable, 2 comfortable, 3 very comfortable; TS, −3 cold, −2 slightly cold, −1 cool, 0 neutral, 1 warm, 2 slightly hot, 3 hot. Values are presented as mean ± SD.

Abbreviation: PWU: Post‐warm‐up.

* *p* < 0.05;

** *p* < 0.01 of HEAT versus CON in the specific environmental condition.

#### 3.2.2 Performance readiness

The Elite Performance Readiness Questionnaire was used to report perceptual responses (Table 2). Immediately following the half‐time warm‐up, following the application of HEAT, players felt less fatigued (*F*_1,13_ = 10.63; *p* = 0.006), less sore (*F*_1,13_ = 5.44; *p* = 0.036), more motivated (*F*_1,13_ = 8.01; *p* = 0.014), more confident (*F*_1,13_ = 9.32; *p* = 0.009), along with feeling an increase in match readiness (*F*_1,13_ = 6.06; *p* = 0.026). Additionally, immediately prior to the SMS at 60 min, players felt less fatigued (*F*_1,13_ = 6.29; *p* = 0.026), less sore (*F*_1,13_ = 6.02; *p* = 0.029), more motivated (*F*_1,13_ = 15.85; *p* = 0.002), more confident (*F*_1,13_ = 8.54; *p* < 0.012), more alert (*F*_1,13_ = 6.73; *p* = 0.022), and had an increased perception of match readiness (*F*_1,13_ = 10.36; *p* = 0.006).

**TABLE 2:** Perceptual responses measured via Elite Performance Readiness Questionnaire, measured Post WU1, Post WU2 and 60 min.

	Post WU1				Post WU2				60 min			
	HEAT18	CON18	HEAT02	CON02	HEAT18	CON18	HEAT02	CON02	HEAT18	CON18	HEAT02	CON02
Fatigue	2.3 ± 0.9	2.8 ± 1.8	2.0 ± 1.1	2.6 ± 1.6	1.9 ± 0.9	2.9 ± 1.7**	1.7 ± 1.2	2.9 ± 1.5**	1.6 ± 1.0	2.8 ± 1.8*	1.5 ± 1.3	3.1 ± 1.8*
Soreness	2.2 ± 0.8	2.6 ± 2.3	1.9 ± 1.3	2.1 ± 1.6	1.9 ± 1.0	3.1 ± 2.3*	1.8 ± 1.0	3.0 ± 2.1*	1.8 ± 1.3	3.2 ± 2.6*	2.0 ± 1.6	3.2 ± 2.0*
Motivation	6.4 ± 1.8	6.4 ± 1.5	6.2 ± 1.8	6.0 ± 1.8	6.5 ± 1.8	6.6 ± 1.5	6.8 ± 1.5	5.3 ± 1.9*	7.0 ± 1.4	6.3 ± 1.6*	6.9 ± 1.4	4.8 ± 1.9**
Anger	0.8 ± 0.9	0.4 ± 0.7	0.5 ± 0.6	0.6 ± 0.7	0.6 ± 0.7	0.3 ± 0.3	0.4 ± 0.7	0.7 ± 1.0	0.7 ± 0.9	0.3 ± 0.3	0.4 ± 0.6	0.7 ± 0.8
Confusion	0.5 ± 0.8	0.4 ± 0.6	0.6 ± 0.7	0.5 ± 0.7	0.8 ± 1.4	0.5 ± 0.5	0.5 ± 0.8	0.7 ± 1.2**	0.5 ± 0.7	0.3 ± 0.4	0.4 ± 0.5	0.7 ± 0.9
Depression	0.5 ± 0.9	0.4 ± 0.9	0.4 ± 0.5	0.4 ± 0.6	0.6 ± 1.0	0.5 ± 0.7	0.4 ± 0.6	0.4 ± 0.6	0.6 ± 1.0	0.4 ± 0.6	0.4 ± 0.6	0.5 ± 0.7
Tension	1.3 ± 1.0	1.4 ± 1.2	1.5 ± 1.4	1.5 ± 1.2	1.4 ± 0.8	2.0 ± 1.7	1.7 ± 1.5	2.2 ± 1.5	1.7 ± 1.6	2.0 ± 2.0	1.5 ± 1.5	2.9 ± 1.9
Alertness	5.8 ± 1.7	5.8 ± 1.6	6.2 ± 1.5	6.1 ± 1.3	6.5 ± 2.0	6.1 ± 1.9	6.4 ± 1.9	5.4 ± 1.9	6.6 ± 1.8	5.9 ± 1.8*	6.3 ± 1.4	5.2 ± 1.9*
Confidence	6.3 ± 1.7	6.2 ± 1.3	6.5 ± 1.7	6.7 ± 1.5	6.6 ± 1.9	6.4 ± 1.7	6.9 ± 1.5	5.9 ± 1.6**	6.6 ± 1.8	6.1 ± 1.5	6.8 ± 1.5	5.7 ± 1.5**
Sleep	5.9 ± 1.6	6.5 ± 1.2	6.2 ± 1.5	5.8 ± 2.0	5.8 ± 1.6	6.1 ± 1.4	6.0 ± 1.9	5.6 ± 1.8	5.6 ± 1.9	6.2 ± 1.2	6.1 ± 1.5	5.7 ± 2.0
Readiness	6.8 ± 1.2	7.0 ± 1.2	7.0 ± 1.1	6.9 ± 1.6	7.3 ± 1.0	7.0 ± 1.6	7.4 ± 0.8	6.4 ± 1.3*	7.6 ± 1.0	7.0 ± 1.5	7.4 ± 1.1	5.7 ± 1.6**

*Note*: Values are mean ± SD. Data presented as mean ± SD.

Abbreviations: WU1: Pre‐match warm‐up, WU2: Half‐time warm‐up.

* *p* < 0.05;

** *p* < 0.01 versus the same time point in HEAT.

### 3.3 Tympanic temperature

**FIGURE 4:**
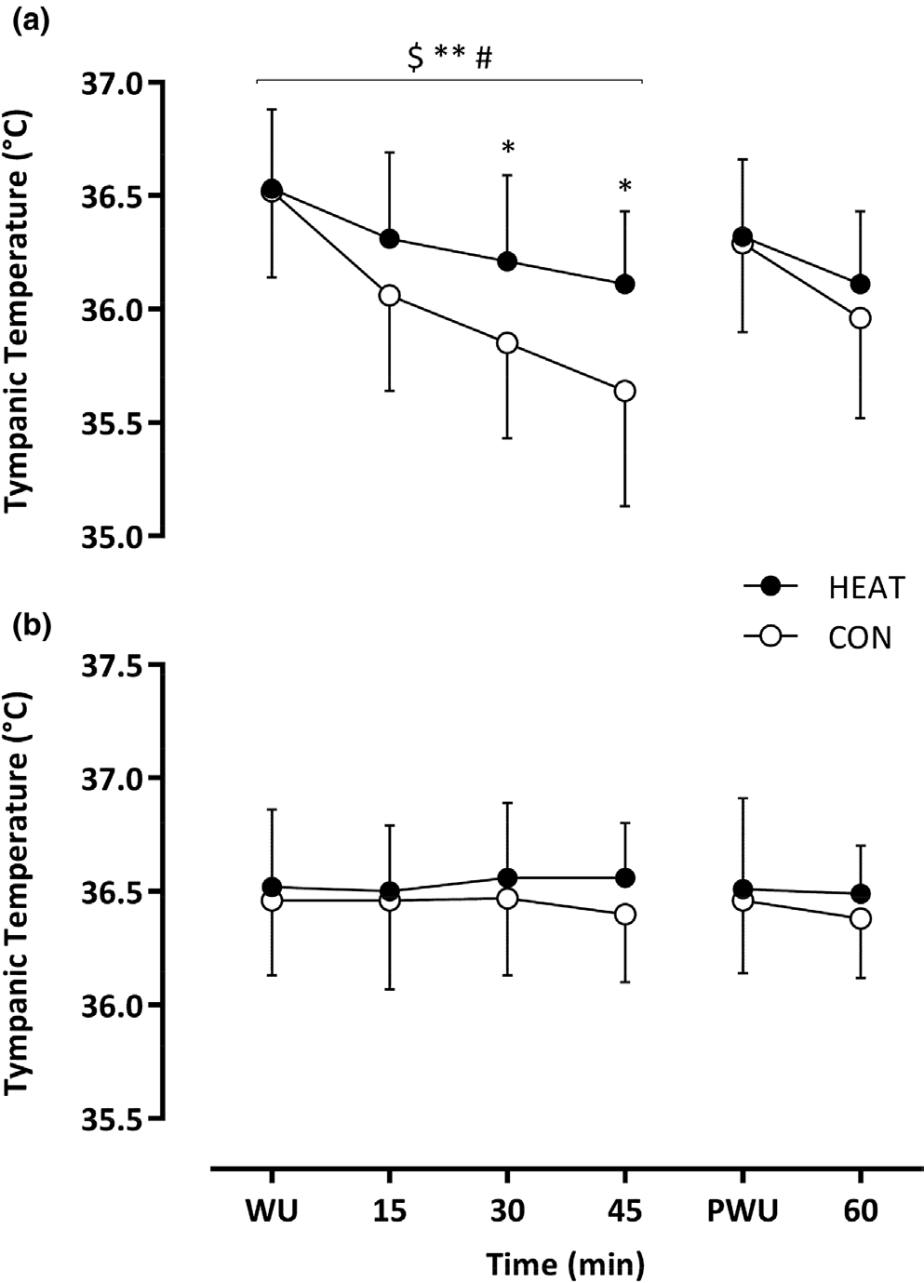
Measurement of tympanic temperature during ambient temperatures of 2°C (a) and 18°C (b) throughout the 60 min of passive recovery. Data presented as mean ± SD. $ denotes a time effect, ** denotes condition effect, and # denotes an interaction effect (*p* < 0.05).

Baseline T_tymp_ was similar in each condition (*p* = 0.95). Throughout the first half, T_tymp_ reduced over time (*F*_3,39_ = 17.46, *p* < 0.001) and the decline was attenuated in HEAT versus CON (Δ –0.4 ± 0.3 vs. Δ −0.9 ± 0.5°C; *F*_1,13_ = 5.17; *p* = 0.041). Furthermore, the rate of change in T_tymp_ differed over time between HEAT versus CON (*F*_3,39_ = 4.54, *p* = 0.025), with post hoc analyses confirming that T_tymp_ was higher in HEAT after 30 (Δ 0.4°C; *p* = 0.26) and 45 min (Δ 0.5°C; *p* = 0.007). After the half‐time warm‐up, T_tymp_ reduced over time (*F*_1,13_ = 5.035, *p* = 0.043) and the decline was comparable in HEAT versus CON (Δ –0.2 ± 0.3 vs. Δ −0.3 ± 0.4°C; *F*_1,13_ = 1.036; *p* = 0.074) (Figure 4).

### 3.4 18°C condition

#### 3.4.1 Performance

Post pre‐match warm‐up, similar baseline measures of CMJ (HEAT: 39.6 ± 4.5 vs. CON: 39.1 ± 5.7 cm; *F*_1,13_ = 0.5; *p* = 0.49) and RSM (HEAT: 4.45 ± 0.23 vs. CON: 4.48 ± 0.24 s; *F*_1,13_ = 0.56; *p* = 0.47) were observed. Throughout the SMS, CMJ (*F*_3,11_ = 13.3, *p* < 0.001) and RSM (*F*_3,11_ = 8, *p* = 0.001) reduced over time but were greater in HEAT versus CON for CMJ (*F*_1,13_ = 6.9, *p* = 0.021) along with an enhanced RSM (*F*_1,13_ = 28.2, *p* < 0.001). Further, the rate of change in CMJ (*F*_3,11_ = 2.25, *p* = 0.14) and RSM (*F*_3,11_ = 2.12, *p* = 0.139) did not differ over time between conditions. Following the 60‐min substitute simulation, post hoc analyses showed no differences in CMJ or RSM (Figure 2b & 3b).

#### 3.4.2 Soccer match simulation

Following the application of HEAT versus CON, throughout the SMS, mean 15 m sprint times improved (2.51 ± 0.14 vs. 2.57 ± 0.13 s; *F*_1,13_ = 26.73; *p* < 0.001). However, 15 m sprint time was not reduced over time. Throughout the SMS, dribbling speed (5.67 ± 0.69 vs. 5.68 ± 0.83 s; *F*_1,13_ = 0.006; *p* = 0.94) and precision (30.43 ± 3.61 vs. 31.03 ± 2.49 cm; *F*_1,13_ = 0.406; *p* = 0.54) were comparable between HEAT and CON. Further, the rate of change in dribbling speed and precision did not differ over time (*p* = 0.204).

### 3.5 Perceptual response

#### 3.5.1 Thermal Comfort and Thermal Sensation

TC and TS were similar at baseline in each condition. Throughout the simulated first half, TC (*F*_3,39_ = 4.95, *p* = 0.033) and TS (*F*_3,39_ = 14.7, *p* < 0.001) reduced over time and the decline was attenuated in HEAT versus CON in TC (*F*_1,13_ = 7.868, *p* = 0.015) and TS (*F*_1,13_ = 25.594, *p* < 0.001). Further, the rate of change differed in TC (*F*_3,39_ = 8.390, *p* = 0.002) and TS (*F*_3,39_ = 3.9, *p* = 0.034) over time between conditions and post hoc analyses confirmed that participants felt “more comfortable” and/or “warmer” after 15 (TS: *p* = 0.007), 30 (TC: *p* = 0.003; TS: *p* = 0.006) and 45 min (TC: *p* = 0.003; TS: *p* = 0.002) in HEAT versus CON. During the simulated second half, TC (*F*_1,13_ = 13, *p* = 0.003) and TS (*F*_1,13_ = 21.311, *p* < 0.001) reduced over time and the decline was attenuated in HEAT versus CON in TC (*F*_1,13_ = 8.412, *p* = 0.012) and TS (*F*_1,13_ = 16.278, *p* = 0.001). Further, the rate of change in TC (*F*_1,13_ = 5.508, *p* = 0.035) and TS (*F*_1,13_ = 6.303, *p* = 0.026) differed over time between conditions and post hoc analyses confirmed that participants felt “more comfortable” and “warmer” after minute 60 (TC: *p* = 0.002; TS: *p* < 0.001) (Table 1).

#### 3.5.2 Performance readiness

Immediately following the half‐time warm‐up, following the application of HEAT, players felt less fatigued (*F*_1,13_ = 6.49; *p* < 0.002) and less sore (*F*_1,13_ = 6.14; *p* = 0.028). Additionally, prior to match entry at 60 min, players felt less fatigued (*F*_1,13_ = 6.29; *p* = 0.026), less sore (*F*_1,13_ = 6.022; *p* = 0.029), and more alert (*F*_1,13_ = 6.73; *p* = 0.022) (Table 2).

### 3.6 Tympanic temperature

Temperature at baseline was similar in each condition (*p* = 0.46). Throughout the 1st half, T_tymp_ was comparable between HEAT and CON (*F*_3,39_ = 0.1, *p* = 0.85) and no change in T_tymp_ was observed between HEAT and CON (Δ0 ± 0.2 vs. Δ − 0.1 ± 0.3°C; *F*_1,13_ = 2.041; *p* = 0.177; interaction effect, *F*_3,39_ = 0.635; *p* = 0.541). After the half‐time warm‐up, T_tymp_ was comparable over time (*F*_1,13_ = 0.360, *p* = 0.559) and no change was detected in HEAT versus CON (Δ 0 ± 0.2 vs. Δ −0.1 ± 0.3°C; *F*_1,13_ = 1.29; *p* = 0.277). Furthermore, T_tymp_ did not differ over time between HEAT versus CON (*F*_1,13_ = 0.222, *p* = 0.646) (Figure 4b).

## 4 DISCUSSION

This study aimed to determine the performance, perceptual and temperature impacts of heated garments for soccer substitutes throughout a closed substitute simulation in both a thermoneutral and cold environment. The main findings of this study were that following the HEAT intervention, throughout the 30‐min SMS protocol, the average 15 m sprint time improved at both 2 and 18°C. Further, in HEAT a significant main effect on trial and time on CMJ height and RSM time was observed throughout the SMS in both 2 and 18°C. However, no difference in dribbling speed or precision was detected in either environmental condition. In terms of perceptual responses, upon simulated match entry, thermal comfort and sensation were improved in both 2 and 18°C and overall match readiness was enhanced in the 2°C condition following HEAT. Therefore, this present study suggests that the use of heated garments positively impacts some key performance indicators for physical performance for soccer substitutes in both thermoneutral and cold environments.

This study demonstrates that a passive heating strategy can optimize the period of limited activity that a soccer substitute experiences before match entry. These extended periods of limited activity post‐warm‐up have been found to decline core temperature in soccer substitutes thereby reducing performance capabilities (Hills et al., 2021). One key factor leading to the improvement in physical performance is the possible improvement in muscle temperature. Despite direct measures of muscle temperature not being measured in the present study, results from Cowper et al. (2023) show how this specific heating intervention can positively influence muscle (vastus lateralis 2 cm depth) and tympanic temperature during periods of passive rest. Thus, given that a similar substitute simulation protocol, along with a similar group of participants was recruited, provides a logical explanation for the performance improvements found within the current study. Furthermore, the observed improvements in CMJ and RSM performance immediately pre‐SMS, are in line with current literature investigating passive heating strategies and team sport performance. In ambient temperatures, Kilduff et al. (2013) reported that following an active warm‐up, the application of a blizzard survival jacket during a 15‐min passive rest period increased CMJ PPO (3.5%) along with an improved RSA (0.7%). Furthermore, when a similar protocol was implemented during 20 min of passive rest, West et al. (2016) reported an increase of 3.2% in CMJ power and a reduced sprint time. Therefore, these studies show that passive heating strategies can act as a practical solution for maintaining the benefits of warm‐up and preventing a decline in physical performance and bodily temperature. Although an improvement in physical performance was apparent, no changes in dribbling performance were observed. This may be because skill‐based drills may be less influenced by the benefits of muscle temperature improvements than movements which involve greater peak muscular activity such as sprinting and jumping (Russell, Benton, & Kingsley, 2011).

Following the application of the heating garments, the hypothesized improvement in muscle temperature before match entry is a vital contributor to enhanced muscle function and overall improvement in exercise performance. The increase in muscle temperature elicit numerous physiological enhancements, such as increased transmission rate of nerve impulses, decreased stiffness of joints and muscles, increased high energy phosphate degradation, glycogenolysis, and a shift in the force‐velocity relationship (Bishop, 2003b). The enhancement in muscle temperature has been found to increase power output which is a key influence in team sports which utilize repeated sprints. However, limited studies have been conducted that utilize passive heat for long‐duration exercise performance (≥5 min) in thermoneutral environments (Bishop, 2003a). This is thought to be down to increased thermoregulatory strain and early attainment of critical core temperature (Fortney et al., 1984; Nadel, 1987). However, Fairbank et al. (2021), found small improvements in high‐intensity running performance during a 23‐min rugby simulation when a knee‐length sub‐suit jacket was utilized for 15 min in “changing room” conditions (~19.9°C) with the active warm‐up and performance taking place in ~12°C. This supports the present study which found no differences in core temperature throughout the passive rest period at 18°C. Therefore, this provides scope for passive heat to be considered at any temperature ≤ 18°C. The present data supports this supposition, as when in a thermoneutral environment, RSM and CMJ were significantly improved pre‐SMS and RSM was improved throughout the SMS. Furthermore, no detriments in performance were experienced post‐SMS, signifying no risk was observed in physical performance while using the heating intervention in the thermoneutral environment.

Although, muscle temperature is perceived to be the “most important” benefit before exercise, “mental readiness” has also been categorized as just as important (Towlson et al., 2013). The measures of perceptual response monitored before match entry at the 60th minute of the match, found that players felt warmer and more comfortable at both 2 and 18°C. Such findings are important as increased perceived comfort can elicit improvements in sporting performance (Schlader et al., 2011). Indeed, being warm causes widespread changes in the central nervous system (Lowry & Nutt, 2009). Therefore, the increased perception of warmth and the benefits of an elevated body temperature following an active warm‐up and the application of the heated trousers may have been attributed to the significant decreases in perceived fatigue and muscle soreness and overall, a rise in readiness to perform in both environmental conditions (McGorm et al., 2018; Wilkins & Havenith, 2017).

### 4.1 Limitations

A limitation of this study was that the performance aspect of the protocol was a modified version of the SMS. The ecological validity of this is less than that of an open‐play soccer match due to it being a set routine constructed from audio cues, whereas an open‐play match would fluctuate intensities and reactions based on external stimuli, which might alter players motivations and physiological capacity (Goodall et al., 2017). However, the distance covered by players were similar to the demands of Premier League players (Bloomfield et al., 2007). The SMS protocol does serve as a valid method to measure the demands of soccer and allows for precise reproducibility between each condition (Briggs et al., 2017; Carling et al., 2016; Russell, Rees, et al., 2011).

Furthermore, the inability to perform a pre‐match warm‐up, half‐time re‐warm‐up, and the SMS in the environmental chamber, therefore, they were performed at room temperature on an indoor sprint track (~18°C). This led to prolonged periods out of the 2°C condition which might have increased the muscle and core temperature at a higher rate than if they were performed in the environmental chamber at 2°C. While this study aimed to emulate the passive intervention period conducted in Cowper et al. (2023), pitch‐side warm‐ups throughout the first half were not implemented. Further work should look to assess the impact of such heat interventions in the field, to understand the direct ecological feasibility of using such intervention strategies. Further, although a significant attenuation in T_tymp_ was observed throughout the 2°C‐condition following HEAT, a limitation of this study was that T_tymp_ was utilized as the measure of core temperature. Due to the ear shell being not well shielded, an underestimation of core temperature may have been observed in the 2°C environmental conditions.

### 4.2 Practical implications

The soccer season is typically played in a variety of temperatures. Particularly in winter conditions, soccer substitutes can experience large reductions in core and muscle temperature, mainly due to their inactivity as a substitute and sub‐optimal clothing (Cowper et al., 2023). The results from the present study indicate that soccer substitutes which apply a passive heating intervention before match entry may perform better physically, in the form of jump and sprint ability in both cold and thermoneutral conditions. Further, players might elicit a higher degree of “readiness” when entering match‐play. This may increase the probability that the substitute performs better, which could affect the overall outcome of a game. These findings may be applicable to other sports where lengthy passive rest periods post warm‐up are experienced, in particular, events which are frequently performed in the cold.

## 5 CONCLUSION

In conclusion, this study demonstrates that the application of heated trousers before match entry for soccer substitutes led to improvements in physical performance, in both thermoneutral and cold conditions throughout the SMS protocol. In addition, improvements in perceptual responses including match readiness, thermal comfort, and sensation give scope for heating trousers to be utilized for soccer substitutes prior to match entry when matches are performed in a range of environmental temperatures <18°C.

## FUNDING INFORMATION

The authors received no financial support for the research, authorship, and/or publication of this article.

## CONFLICT OF INTEREST STATEMENT

The authors declare no conflicts of interest.

## ETHICS STATEMENT

The study was approved by Northumbria University ethics committee (#45401) and all procedures were conducted according to all aspects of the Declaration of Helsinki, apart from registration in a database.

## Data Availability

The data that support the findings of this study are available on request from the corresponding author, GC.
